# Supplementary material for: CTTN Overexpression Confers Cancer Stem Cell-like Properties and Trastuzumab Resistance via DKK-1/WNT Signaling in HER2 Positive Breast Cancer
Source: Cancers (Basel). 2023 Feb 11;15(4):1168. doi: 10.3390/cancers15041168 (PMC9954024; doi:10.3390/cancers15041168)
Supplement: Supplementary file 1 [file cancers-15-01168-s001.zip › cancers-2194204-supplementary.pdf]

cancers MDPI

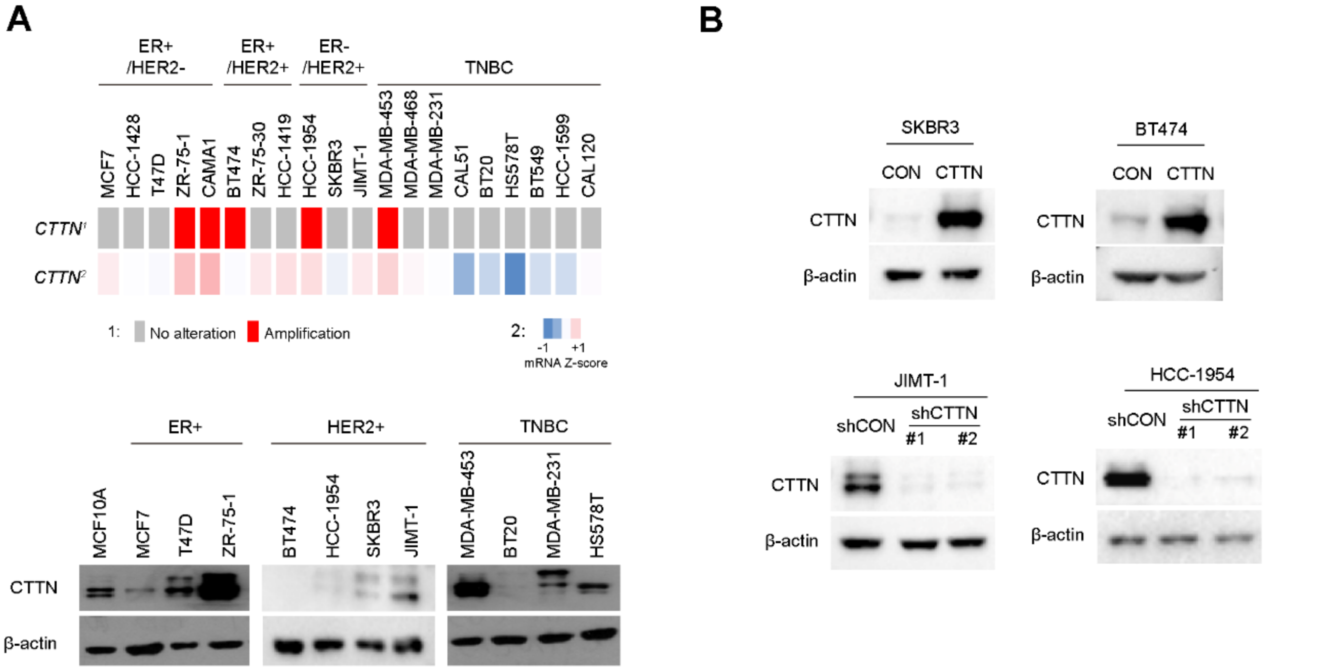

Figure S1. Expression patterns and genetic alterations of *CTTN* in human breast cancer cell lines. (**A**) Oncoprints from the Cancer Cell Line Encyclopedia (CCLE) dataset displaying relative levels of expression and genetic alterations of *CTTN* in several breast cancer cell lines with indicated subtypes (top). Immunoblots showing the expression levels of CTTN in the indicated breast cancer cell lines. **B)** Immunoblots confirming the extents of CTTN overexpression or knockdown in the indicated cell lines.

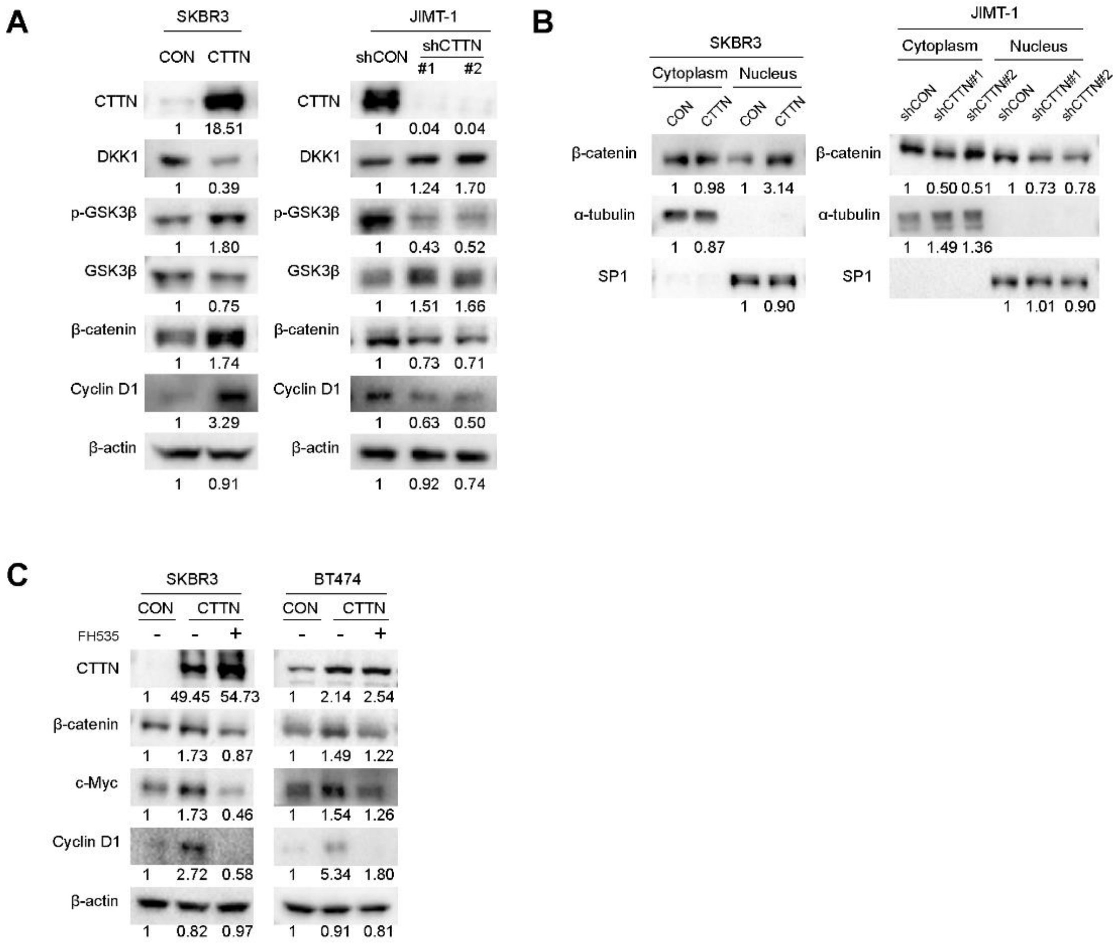

Figure S2. Quantification of western blots using imageJ software. (**A, B and C)** Relative band intensities of western blots in Figure 3E, G, and Figure 4A, respectively. Numbers below each band indicate the relative intensity of the band. All band intensities were normalized with the intensity of the loading controls. Loading control refers to β-actin in Figures S2A and S2C, while α-tubulin and SP1 were used as loading controls for cytoplasmic and nuclear protein samples, respectively, in Figure S2B.

Table S1. List of upregulated and downregulated genes associated with trastuzumab resistance used in heatmap of Figure 3D.

Upregulated trastuzumab resistance-associated genes

No.	Gene	Locus	log2(fold_change)	p-value
1	HIST1H4B	6:26026814-26027252	2.08711	0.02255
2	HPGD	4:174490176-174523154	1.77178	0.00005
3	PSORS1C2	6:31114749-31140092	1.72969	0.0333
4	C2CD4A	15:62066976-62070917	1.61387	0.00005
5	CD36	7:80312573-80679277	1.50663	0.00005
6	GPR162,P3H3	12:6821544-6839851	1.47835	0.00005
7	CACNG1	17:67044589-67056797	1.46698	0.00875
8	HIST1H3A	6:26020489-26020900	1.37422	0.04105
9	DMKN	19:35497219-35513658	1.31764	0.00005
10	FAM133A	X:93674012-93712274	1.27885	0.0002
11	CTTN	11:70270699-70436584	1.27088	0.00005
12	GTSF1	12:54353660-54548238	1.26892	0.0077
13	HIST2H4A	1:149832658-149841193	1.24874	0.00005
14	TPRG1	3:188941714-189325304	1.23833	0.0002
15	DPP10	2:114442298-115845752	1.23417	0.01535
16	KRT13	17:41500980-41505705	1.21609	0.0013
17	SPTBN4	19:40466240-40576464	1.09824	0.01215
18	ADAMTS3	4:72280968-72569386	1.09682	0.00975
19	ITPKA	15:41493392-41503551	1.0617	0.00005
20	LINC01315	22:42364399-42369236	1.04981	0.00005
21	CCDC74B	2:130129620-130145134	1.02569	0.0103
22	ANXA9	1:150982016-150995634	1.01964	0.00005
23	PCP4	21:39867316-39929397	1.00182	0.00005
24	ANKRD29	18:23598925-23662885	0.980893	0.00005
25	SLC30A4	15:45430528-45586304	0.938334	0.0058
26	BOC	3:113211002-113441610	0.921363	0.0099
27	C1orf61	1:156404249-156430701	0.911688	0.0142
28	LMF1	16:853633-981596	0.898651	0.0077
29	CCDC74A	2:131527674-131533666	0.801089	0.00025
30	IL22RA2	6:137143819-137173648	0.794666	0.003
31	SLC7A2	8:17497087-17570573	0.75828	0.0002
32	NMNAT3	3:139389814-139678017	0.701521	0.01065
33	RND2	17:43025240-43032036	0.681158	0.0227
34	GMDS	6:1623805-2245692	0.663014	0.0005

Downregulated trastuzumab resistance-associated genes

No.	Gene	Locus	log2(fold_change)	p_value
1	HS6ST3	13:96090838-96839562	-1.63181	0.00005
2	RIMKLA	1:42380794-42422578	-1.4762	0.0093
3	GPRC5A	12:12890781-12917937	-1.37651	0.00005
4	SLC44A5	1:75202130-75611116	-1.35039	0.00005
5	TAS2R5	7:141790216-141791367	-1.28988	0.00405
6	TRANK1	3:36826819-36945098	-1.2453	0.0499
7	CRISP3	6:49727383-49744437	-1.10123	0.00005
8	CTPS2	X:16588002-16712936	-1.01606	0.00385
9	CA12	15:63321377-63382161	-0.999982	0.00005
10	CYP4Z2P	1:46843094-46900437	-0.947171	0.00005
11	INSR	19:7112254-7294034	-0.938271	0.00075
12	UTRN	6:144285700-144853034	-0.926427	0.0036
13	EHF	11:34621092-34661057	-0.92233	0.00005
14	ENAH	1:225486834-225653142	-0.88005	0.00615
15	NRXN3	14:78170372-79868290	-0.8567	0.00005
16	ALCAM	3:105366908-105576900	-0.823549	0.00005
17	LCN2	9:128149070-128153455	-0.805778	0.00005
18	BPIFB1	20:33273479-33309878	-0.803906	0.00015
19	MAST4	5:66596360-67169595	-0.790917	0.0189
20	AQP3	9:33441153-33447633	-0.783109	0.00005
21	DKK1	10:52314295-52318042	-0.781133	0.01565
22	PGM2L1	11:74330317-74486051	-0.770818	0.00015
23	HOTAIR	12:53962307-53977643	-0.763655	0.00335
24	GPD2	2:156435289-156613735	-0.753186	0.00005
25	ULBP3	6:150063149-150069095	-0.748546	0.00045
26	EPM2A	6:145382534-145737218	-0.722025	0.00375
27	ZNF891	12:133079837-133130473	-0.719246	0.042
28	SAMD4A	14:54567096-54793315	-0.699721	0.00205
29	NUP153	6:17615034-17707344	-0.685619	0.00005
30	CASP2	7:143288214-143307696	-0.682996	0.00005
31	SLC2A13	12:39626166-40106089	-0.680462	0.0012
32	GEN1	2:17663811-17800242	-0.67788	0.01135
33	ARID3B	15:74541176-74598131	-0.677589	0.0067
34	KLF7	2:207074136-207167267	-0.667	0.00075

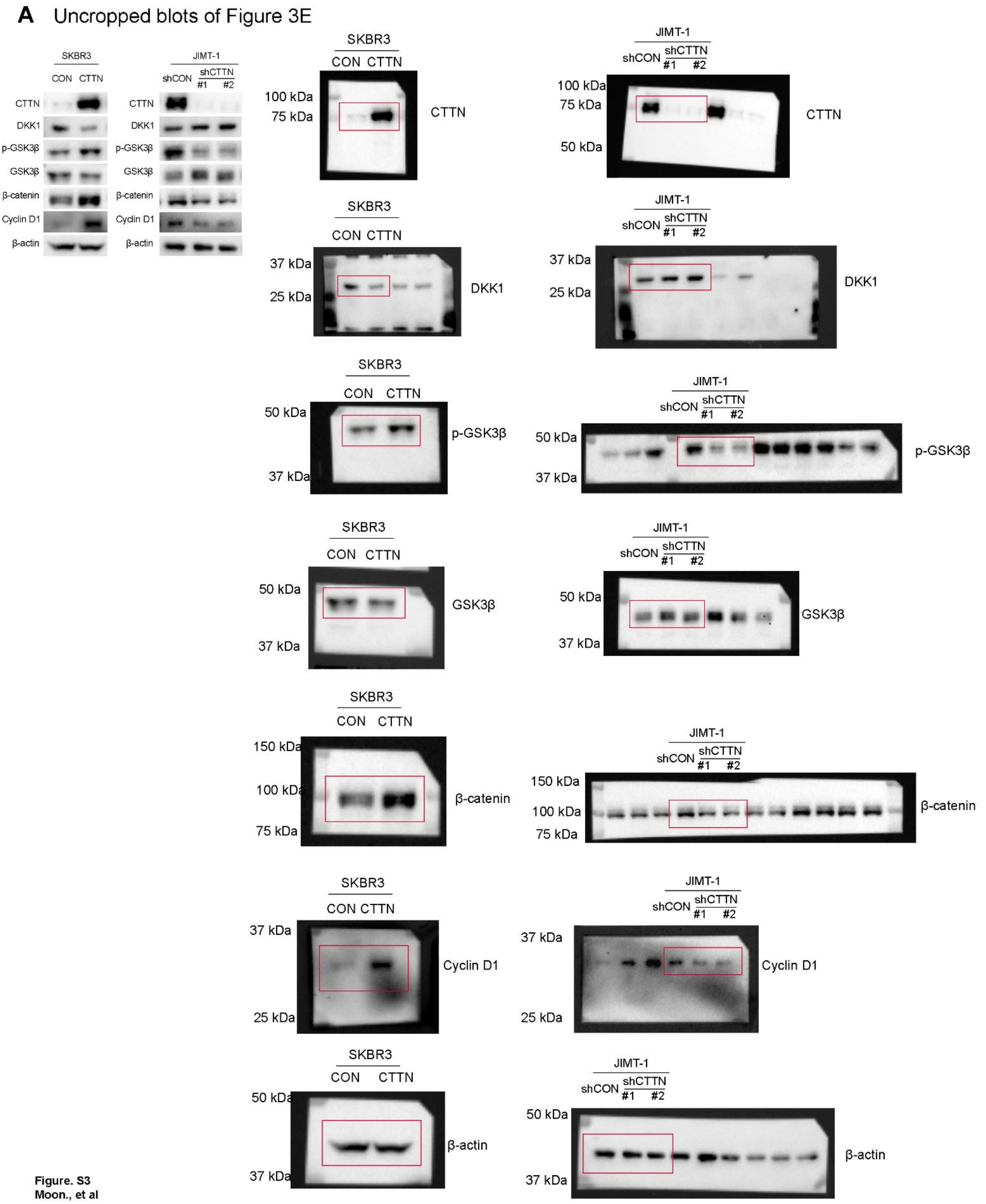

Figure. S3
Moon., et al

B Uncropped blots of Figure 3G

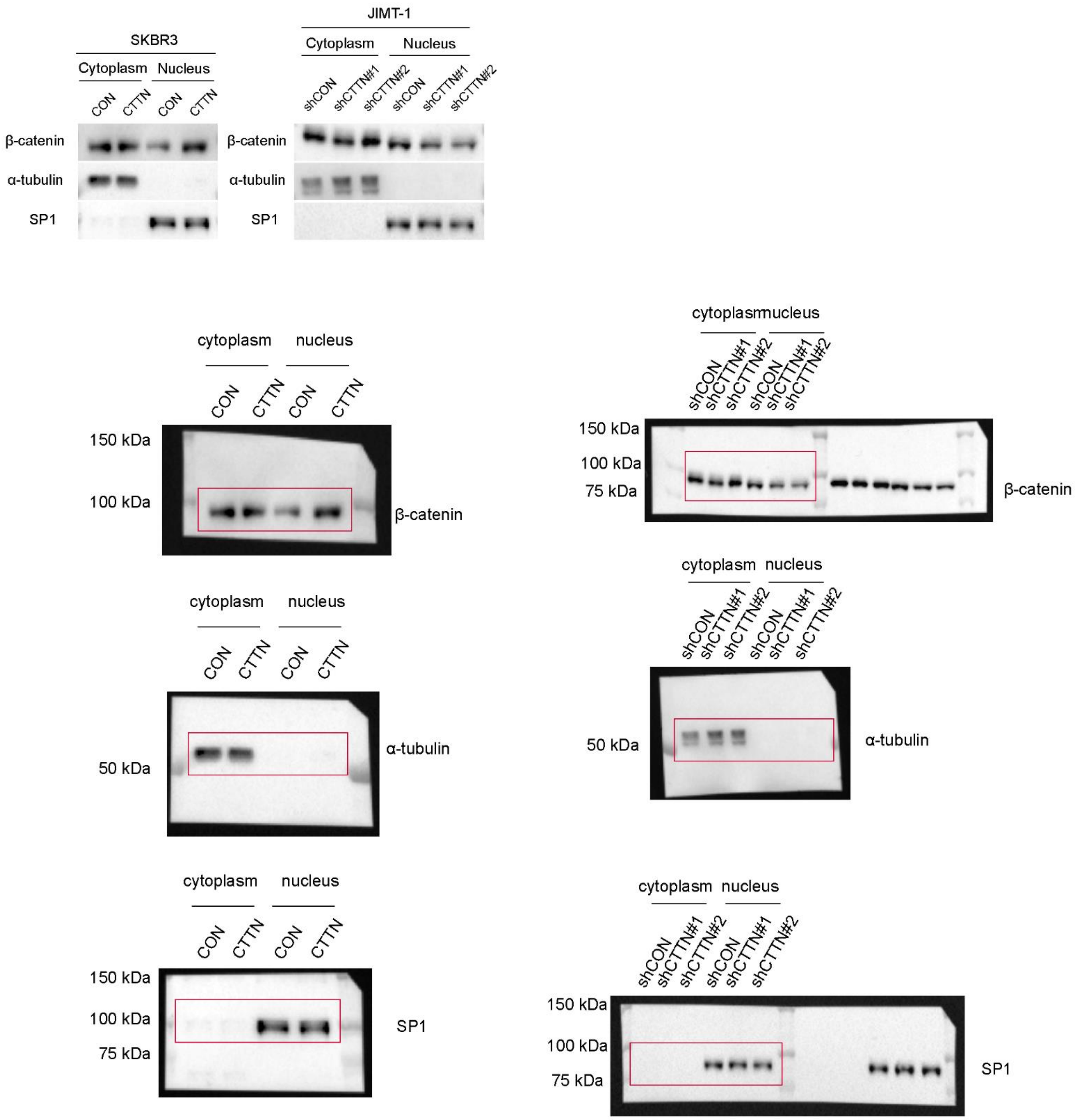

Figure. S3
Moon., et al

C Uncropped blots of Figure 4A

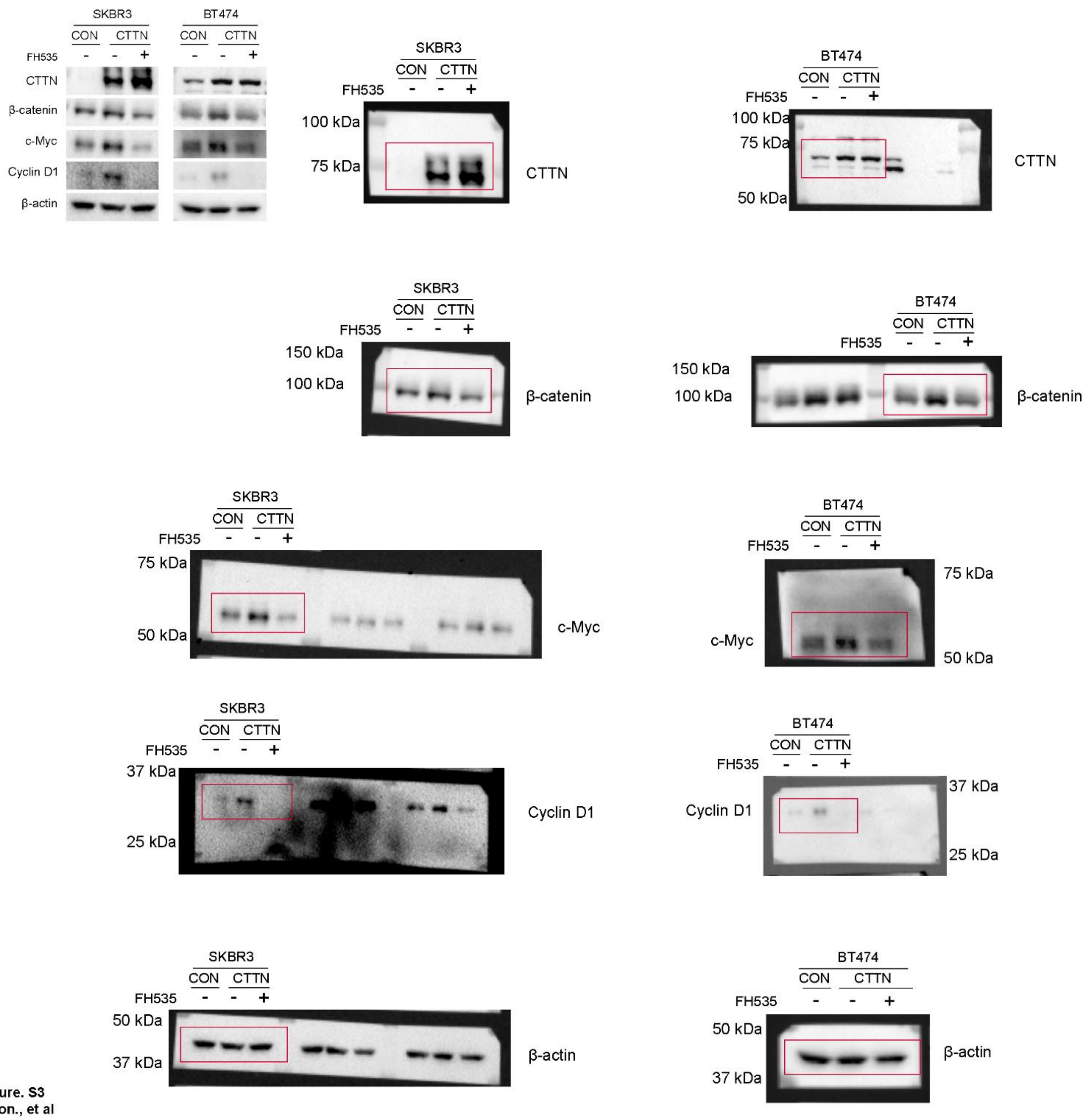

Figure. S3
Moon., et al

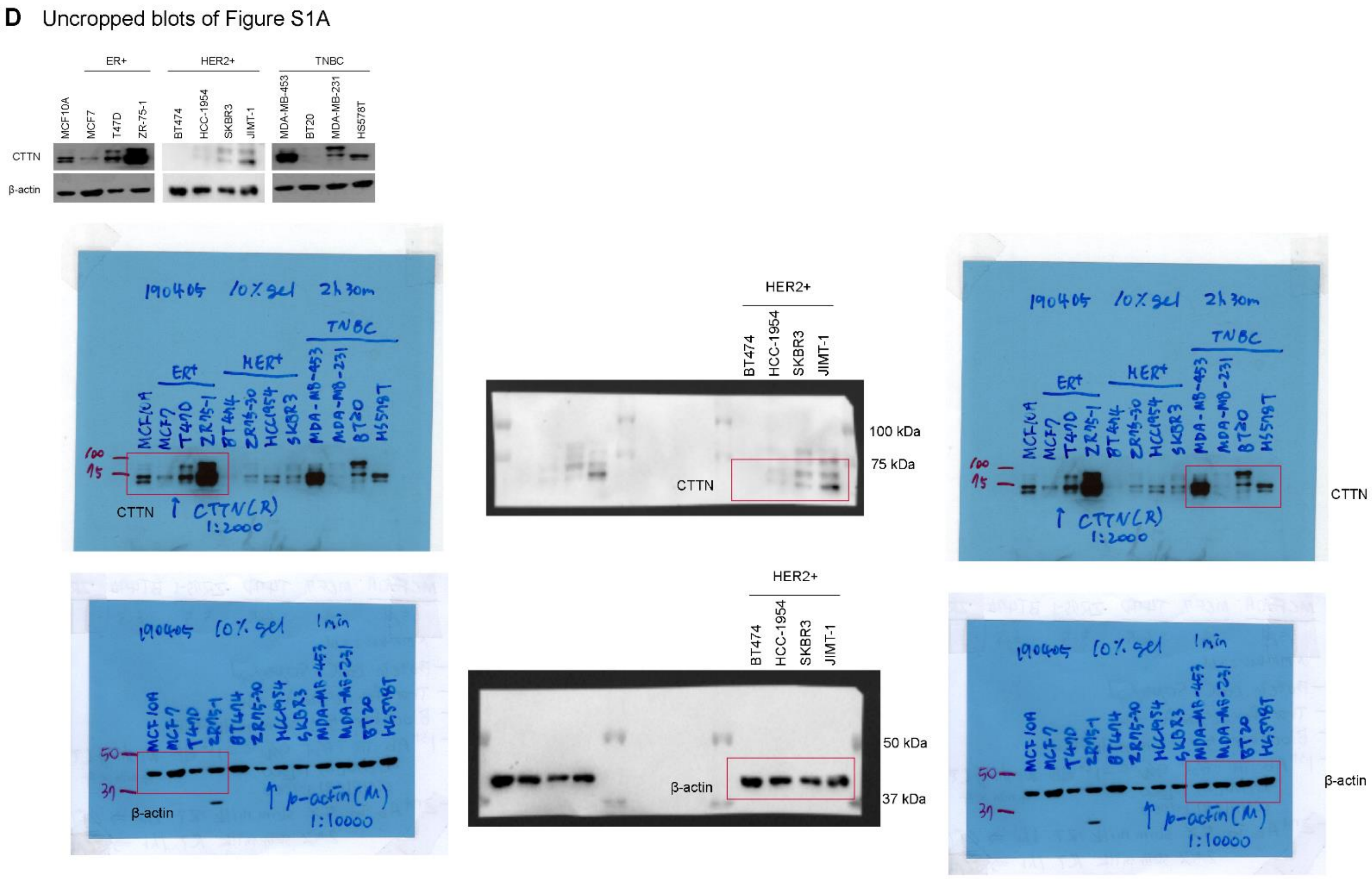

Figure. S3
Moon., et al

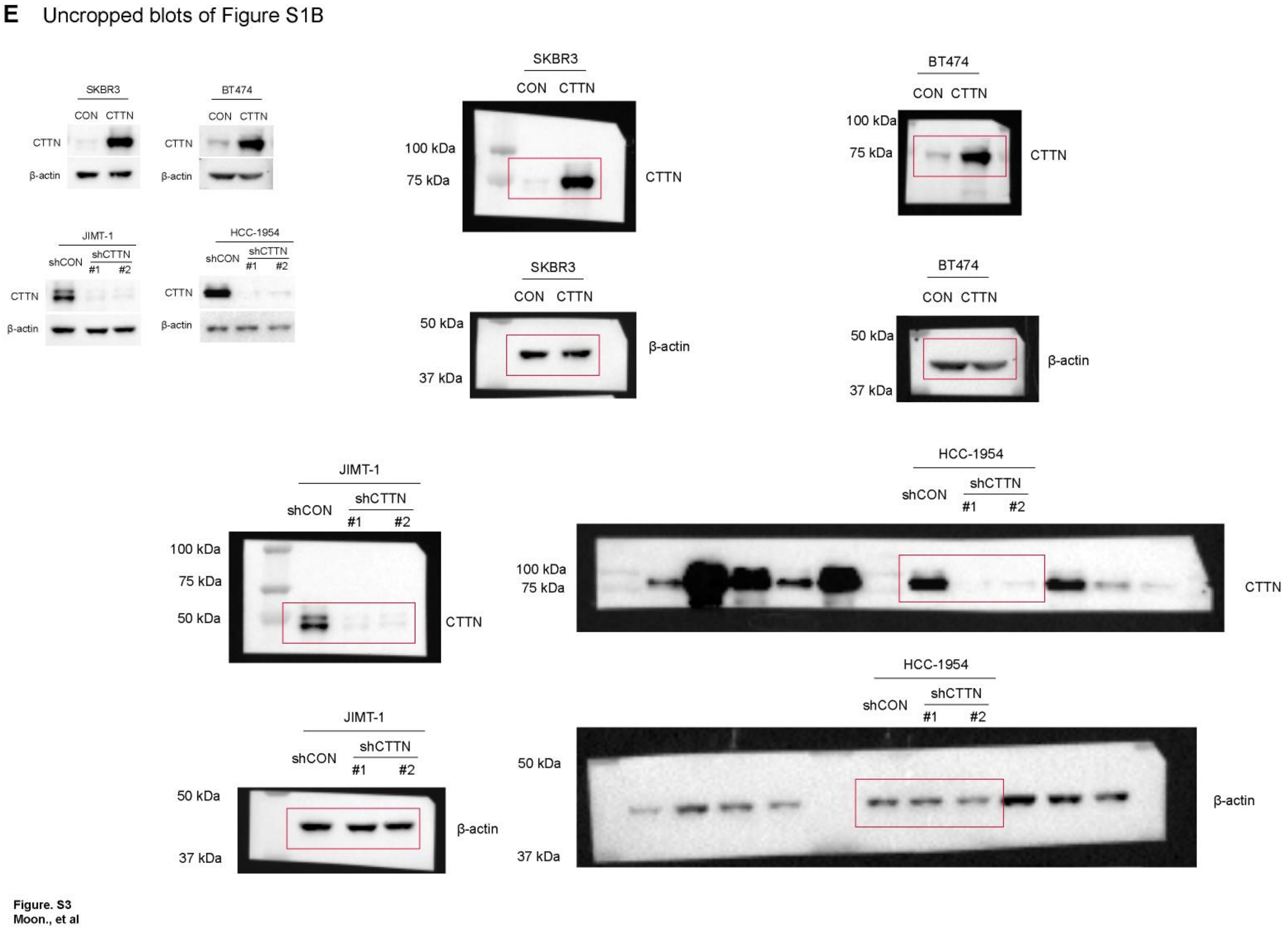

Figure S3. Uncropped western blot images. **(A)** Uncropped western blot images of Figure 3E. **(B)** Uncropped western blot images of Figure 3G **(C)** Uncropped western blot images of Figure 4A. **(D)** Uncropped western blot images of Figure S1A. **(E)** Uncropped western blot images of Figure S1B.
